# A Novel Daratumumab-Based Regimen for Desensitization in Highly HLA-Presensitized Patients Awaiting Kidney Transplantation

**DOI:** 10.3389/ti.2023.11771

**Published:** 2023-08-22

**Authors:** Daqiang Zhao, Zhiliang Guo, Guangyuan Zhao, Rula Sa, Lan Zhu, Gang Chen

**Affiliations:** ^1^ Institution of Organ Transplantation, Tongji Hospital, Tongji Medical College, Huazhong University of Science and Technology, Wuhan, China; ^2^ Key Laboratory of Organ Transplantation, Ministry of Education, Ministry of Public Health, Chinese Academy of Medical Sciences, Wuhan, China

**Keywords:** desensitization, presensitized kidney transplantation, daratumumab, anti-CD38 monoclonal antibody, plasma cells

Dear Editors,

Pre-transplant desensitization is a common strategy to increase the success of renal transplantation in human leukocyte antigen (HLA)-presensitized patients with uremia. Currently, the most efficacious desensitization strategy is to start with rounds of plasmapheresis (PP)/immunoadsorption (IA) together with intravenous immunoglobulin (IVIG) or rituximab [1, 2]. The representative regimens include a combination of rituximab and high-dose IVIG (2 g/kg over 2–4 days) [3], and a combination of 3–5 sessions of PP followed after each session by an infusion of low-dose IVIG (0.1 g/kg) [4]. However, they have limited effectiveness in highly presensitized patients [5]. Daratumumab, an anti-CD38 monoclonal antibody, may be effective in reducing preformed antibodies and desensitizing these patients, given its effect on plasma cell elimination [6]. Two clinical trials are currently investigating daratumumab for desensitization before presensitized kidney transplantation. The first one is the COMBAT trial, in which patients with calculated panel-reactive antibody (cPRA) > 99% will be treated with belatacept in step I, and if ineffective, with four sessions of apheresis, followed by daratumumab monotherapy in step II (ClinicalTrials.gov ID NCT05145296). The second one is the DARDAR trial, in which they investigate daratumumab dose escalation (4–8–16 mg/kg) in step 1 and full dose (16 mg/kg) in step 2 (ClinicalTrials.gov ID NCT04204980). In the literature, cases concerning to daratumumab for pre-transplant desensitization are limited to sensitized heart [7–9] and lung [10] transplant candidates. This letter reports on a case of high HLA sensitization in which a novel daratumumab-based therapy effectively reduced the degree of sensitization and resulted in successful kidney transplantation.

A 32-year-old man (50 kg) was waitlisted for a third kidney transplant. The single-antigen bead assay revealed 22 positive antibodies against HLA-I loci and 14 against HLA-II loci in his serum. Among them, four HLA-I and seven HLA-II antibodies had a high mean fluorescence intensity (MFI) value (>10,000). His cPRA was >99%. With the informed consent, the patient received a new regimen for desensitization to increase the transplant chance.

The treatment was initiated with 200 mg of rituximab. Five days later, PP/IVIG (15–20 g) plus daratumumab (400 mg, intravenously 1 day after PP/IVIG) was given weekly for 19 weeks as an intensive therapy (phase 1, Figure 1A). After phase 1, nine HLA-I and seven HLA-II antibodies were reduced (MFI 5,000–10,000 to 1,401–4,827 and >10,000 to <3,000, respectively) and the other seven HLA-II antibodies became negative (MFI < 1,000) (Figure 1B). The total cPRA decreased from 99.6% to 65.3% and class II cPRA decreased from 98.7% to 0 (MFI cutoff: 5,000, Figure 1C, D).

**FIGURE 1:**
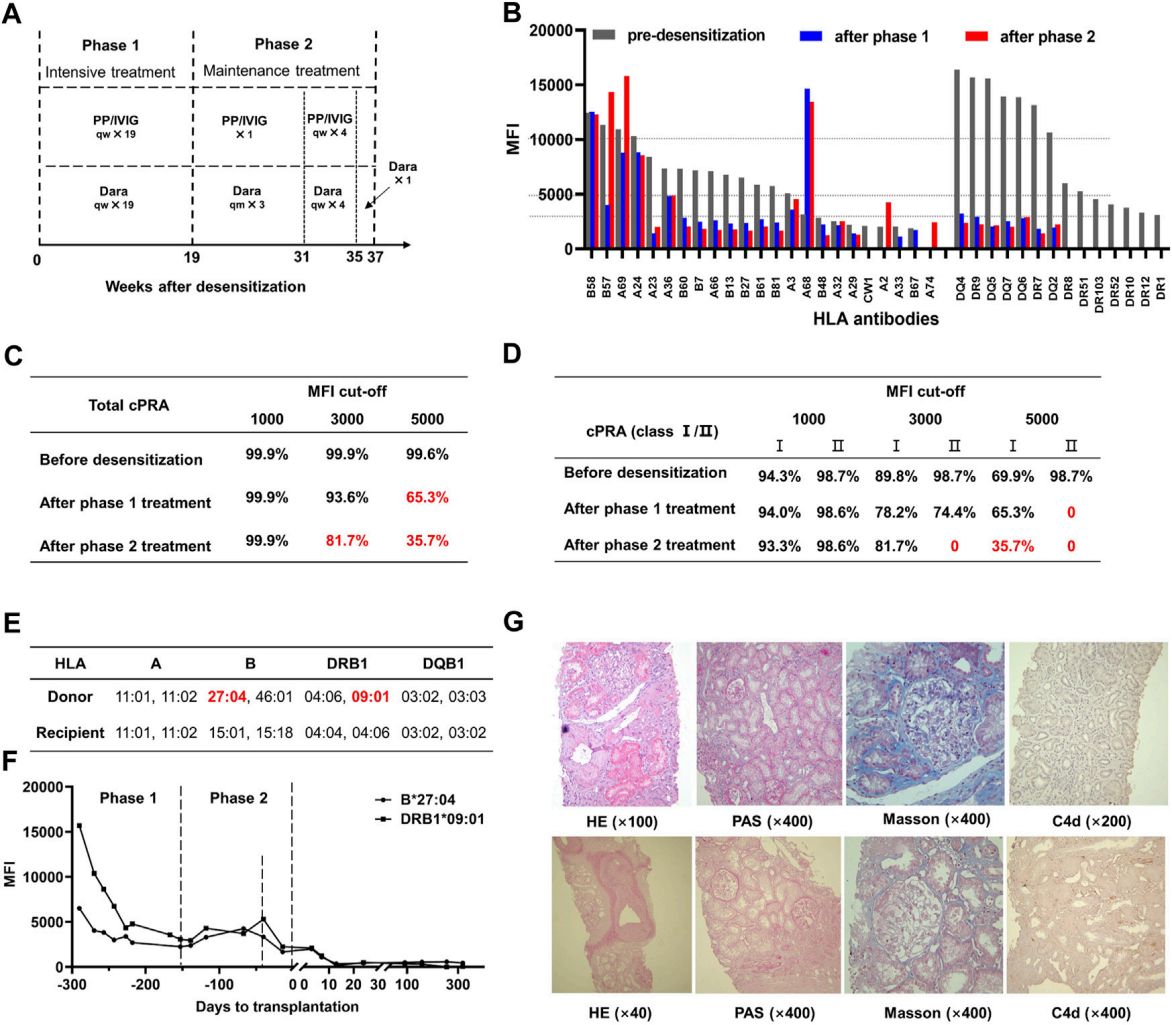
Daratumumab-based HLA desensitization regimen and therapeutic outcomes. **(A)** The desensitization protocol was initiated with 200 mg rituximab. Five days later, plasmapheresis (PP), intravenous immunoglobulin (IVIG, 15–20 g each time) plus daratumumab (Dara, 400 mg, intravenously 1 day after PP/IVIG) were started, with frequencies as indicated. **(B)** Serum HLA antibody levels (1:4 dilution) at various stages. MFI: mean fluorescence intensity. **(C)** Total calculated panel-reactive antibody (cPRA) levels after indicated MFI cutoffs. **(D)** HLA class I/II cPRA levels at the indicated MFI cutoffs. **(E)** Donor-recipient HLA phenotyping indicated two of the preformed antibodies against HLA were donor-specific antibodies (DSA), anti-DRB1 09:01 and anti-B27:04. MM: mismatch. **(F)** DSA levels were significantly decreased after desensitization treatment and did not rebound after transplantation. **(G)** Protocol biopsy results at 3 months (top panel, Banff 2019: i0, t0, g0, v0, ptc0, MVI = 0, ci2, ct1, cg0, cv2, ah2, mm0, Ti0, i-IFTA0, t-IFTA0, C4d0) and 12 months (bottom panel, Banff 2019: i0, t0, g0, v0, ptc0, MVI = 0, ci0, ct0, cg0, cv2, ah2, mm0, Ti0, i-IFTA0, t-IFTA0, C4d1) post-transplantation.

A phase 2 therapy was initiated to maintain the antibody reduction achieved in phase 1 while the patient waited for an HLA-suitable donor. The patient received 400 mg daratumumab monthly and one session of PP/IVIG during the first 12 weeks. Intensive therapy with PP/IVIG plus daratumumab weekly was given to reverse the mild antibody rebound for the following 4 weeks. Daratumumab (400 mg) was administered once during the last 2 weeks (Figure 1A). Desensitization was thus maintained and improved during the 18 weeks after phase 1 (Figure 1B). The total cPRA further decreased from 65.3% to 35.7% and class II cPRA remained at 0 (MFI cutoff: 5,000, Figure 1C, D).

At the end of phase 2, an ABO-compatible kidney obtained from a 52-year-old man after brain death was allocated to the patient via the Chinese organ transplant responding system. The donor-recipient HLA mismatch grade was 4 (Figure 1E). There were low levels of anti- DRB1 0901 (MFI: 2,253) and anti- B2704 (MFI: 1,673) donor-specific antibodies (DSAs) in the patient’s pre-transplant serum, whereas their MFI values before desensitization had been 15,684 and 6,513, respectively (Figure 1F). The complement-dependent cytotoxicity test result was negative.

The allocated kidney was transplanted. The patient received induction therapy of 200 mg rituximab on day −1, 500 mg methylprednisolone daily on days 0–2 and 25 mg thymoglobulin daily on days 0–5 plus maintenance therapy with oral tacrolimus (trough level 7–10 ng/mL), mycophenolate mofetil (750 mg/12 h) and prednisone (10 mg/d). In addition, 20 g/d IVIG on days 1–5 and 15 g/d on days 6–13 were given to prevent DSA rebound. The patient showed good post-operative recovery without any episodes of rejection. DSAs disappeared 2 weeks post-transplantation (Figure 1F). The patient was discharged 30 days after transplantation with a serum creatinine level of 162 μmol/L. Oral compound sulfamethoxazole and valganciclovir were given every 3 days for 3 months to prevent opportunistic infection.

The patient was followed up for 1 year during which graft function remained stable. A 3-month protocol biopsy showed no signs of antibody or T cell-mediated rejections. One-year protocol biopsy results were similar but showed mild peritubular capillary C4d deposition (Figure 1G). DSA levels kept negative, while almost all non-DSAs with high levels before transplantation also declined during the 1-year follow-up period and none of those with low levels rebounded after transplantation.

The patient developed some flu-like symptoms, including nasal discharge, dry cough, and fatigue, after the initial daratumumab administration and was given intravenous infusion of 5 mg dexamethasone in advance of subsequent treatments which suppressed the symptoms. The patient developed myelosuppression during the later stages of intensive desensitization therapy. Myelosuppression was relieved during maintenance treatment, possibly due to the reduced frequency of daratumumab treatment. In addition, no significant toxicity to vision, peripheral nerves or liver was observed and no incidents of infection occurred.

High levels of preformed circulatory antibodies generally take a long time to gradually decay without assistance and the expectation was that the antibody-lowering effect of daratumumab monotherapy might be difficult to show in the short term. However, PP directly removed some of the circulating antibodies and IVIG reduced endogenous antibody rebound after PP-mediated depletion. Therefore, the regime was designed to elicit a synergistic action of daratumumab and PP/IVIG in reducing antibodies and improving desensitization. When a significant response to the intensive desensitization therapy had been achieved, phase 2 treatment with daratumumab monotherapy was initiated to maintain the antibody reduction while waiting for an HLA-matched donor.

In conclusion, the present case provides a novel desensitization strategy for kidney transplantation in highly presensitized patients. Further clinical studies are required for validation of this approach.

## Data Availability

The original contributions presented in the study are included in the article/supplementary material, further inquiries can be directed to the corresponding authors.

## Abbreviations

cPRA, calculated panel-reactive antibody; DSA, donor-specific antibody; HLA, human leukocyte antigen; IVIG, intravenous immunoglobulin; MFI, mean fluorescence intensity; PP, plasmapheresis.

## References

[1] Mamode N Bestard O Claas F Furian L Griffin S Legendre C European Guideline for the Management of Kidney Transplant Patients With HLA Antibodies: By the European Society for Organ Transplantation Working Group. Transpl Int (2022) 35:10511. 10.3389/ti.2022.10511 36033645 PMC9399356

[2] Schinstock C Tambur A Stegall M. Current Approaches to Desensitization in Solid Organ Transplantation. Front Immunol (2021) 12:686271. 10.3389/fimmu.2021.686271 34046044 PMC8144637

[3] Vo AA Lukovsky M Toyoda M Wang J Reinsmoen NL Lai CH Rituximab and Intravenous Immune Globulin for Desensitization During Renal Transplantation. N Engl J Med (2008) 359:242–51. 10.1056/NEJMoa0707894 18635429

[4] Montgomery RA Lonze BE King KE Kraus ES Kucirka LM Locke JE Desensitization in HLA-Incompatible Kidney Recipients and Survival. N Engl J Med (2011) 365:318–26. 10.1056/NEJMoa1012376 21793744

[5] Kozlowski T Andreoni K. Limitations of Rituximab/IVIg Desensitization Protocol in Kidney Transplantation; Is This Better Than a Tincture of Time? Ann Transpl (2011) 16:19–25. 10.12659/aot.881860 21716181

[6] Joher N Matignon M Grimbert P. HLA Desensitization in Solid Organ Transplantation: Anti-CD38 to Across the Immunological Barriers. Front Immunol (2021) 12:688301. 10.3389/fimmu.2021.688301 34093594 PMC8173048

[7] Kwun J Matignon M Manook M Guendouz S Audard V Kheav D Daratumumab in Sensitized Kidney Transplantation: Potentials and Limitations of Experimental and Clinical Use. J Am Soc Nephrol (2019) 30:1206–19. 10.1681/ASN.2018121254 31227636 PMC6622431

[8] Jordan S Vescio R Ammerman N Toyoda M Ge S Chu M Daratumumab for Desensitization and Antibody Mediated Rejection Treatment in Highly-HLA Sensitized Patients. Am J Transpl (2020) 20(suppl 3). Available at: https://atcmeetingabstracts.com/abstract/daratumumab-for-desensitization-and-antibody-mediated-rejection-treatment-in-highly-hla-sensitized-patients/ (Accessed August 16, 2023).

[9] Curtis A Guha A Bhimaraj A Kim J Suarez E Trachtenberg B Use of Daratumumab for Desensitization Prior to Cardiac Transplantation: A Case Report. J Heart Lung Transpl (2021) 40(Suppl):S493. 10.1016/j.healun.2021.01.2015

[10] Magda G Ramsey AL Saggar R Shino MY Weigt SS Reed EF Daratumumab for Desensitization Therapy in Lung Transplant Candidates. J Heart Lung Transpl (2021) 40(Suppl):S335. 10.1016/j.healun.2021.01.943

